# Supplementary material for: 3D-Printed Poly (P-Dioxanone) Stent for Endovascular Application: In Vitro Evaluations
Source: Polymers (Basel). 2022 Apr 26;14(9):1755. doi: 10.3390/polym14091755 (PMC9103802; doi:10.3390/polym14091755)
Supplement: Supplementary file 1 [file polymers-14-01755-s001.zip › polymers-1651615-supplementary.pdf]

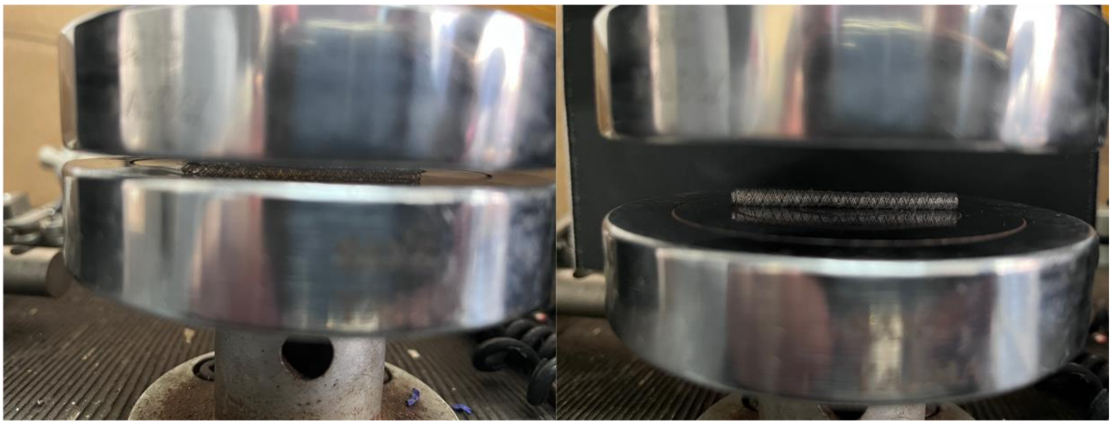

Figure S1. Photos of BRS compressing to 50% diameter and recover to initial diameter after unloading compression for 30s.
